# NH125 Sensitizes Staphylococcus aureus to Cell Wall-Targeting Antibiotics through the Inhibition of the VraS Sensor Histidine Kinase

**DOI:** 10.1128/spectrum.04861-22

**Published:** 2023-05-25

**Authors:** Shrijan Bhattarai, Lane Marsh, Kelsey Knight, Liaqat Ali, Antonio Gomez, Allison Sunderhaus, May H. Abdel Aziz

**Affiliations:** a Fisch College of Pharmacy, The University of Texas at Tyler, Tyler, Texas, USA; Riverside University Health System, Medical Center -University of California

**Keywords:** NH125, *Staphylococcus aureus*, VraS, antibiotic resistance

## Abstract

Staphylococcus aureus utilizes the two-component regulatory system VraSR to receive and relay environmental stress signals, and it is implicated in the development of bacterial resistance to several antibiotics through the upregulation of cell wall synthesis. VraS inhibition was shown to extend or restore the efficacy of several clinically used antibiotics. In this work, we study the enzymatic activity of the VraS intracellular domain (GST-VraS) to determine the kinetic parameters of the ATPase reaction and characterize the inhibition of NH125 under *in vitro* and microbiological settings. The rate of the autophosphorylation reaction was determined at different GST-VraS concentrations (0.95 to 9.49 μM) and temperatures (22 to 40°C) as well as in the presence of different divalent cations. The activity and inhibition by NH125, which is a known kinase inhibitor, were assessed in the presence and absence of the binding partner, VraR. The effects of inhibition on the bacterial growth kinetics and gene expression levels were determined. The GST-VraS rate of autophosphorylation increases with temperature and with the addition of VraR, with magnesium being the preferred divalent cation for the metal-ATP substrate complex. The mechanism of inhibition of NH125 was noncompetitive in nature and was attenuated in the presence of VraR. The addition of NH125 in the presence of sublethal doses of the cell wall-targeting antibiotics carbenicillin and vancomycin led to the complete abrogation of Staphylococcus aureus Newman strain growth and significantly decreased the gene expression levels of *pbpB*, *blaZ*, and *vraSR* in the presence of the antibiotics.

**IMPORTANCE** This work characterizes the activity and inhibition of VraS, which is a key histidine kinase in a bacterial two-component system that is involved in Staphylococcus aureus antibiotic resistance. The results show the effect of temperature, divalent ions, and VraR on the activity and the kinetic parameters of ATP binding. The value of the *K_M_* of ATP is vital in designing screening assays to discover potent and effective VraS inhibitors with high translational potential. We report the ability of NH125 to inhibit VraS *in vitro* in a noncompetitive manner and investigate its effect on gene expression and bacterial growth kinetics in the presence and absence of cell wall-targeting antibiotics. NH125 effectively potentiated the effects of the antibiotics on bacterial growth and altered the expression of the genes that are regulated by VraS and are involved in mounting a resistance to antibiotics.

## INTRODUCTION

Methicillin-resistant and vancomycin-resistant Staphylococcus aureus (S. aureus) are major sources of lethal infections. Some of these strains are showing resistance to recently introduced antibiotics that are thought to be the “last line of defense,” such as daptomycin and teicoplanin (1–3). Several key bacterial systems are involved in driving antibiotic resistance. S. aureus utilizes the two-component system (TCS) VraSR (VraS histidine kinase and VraR response regulator) to receive and relay cell wall-related environmental stress signals. VraSR is highly implicated in the development of resistance through upregulating cell wall synthesis gene clusters after exposure to antibiotics (4, 5). Multiple reports highlighted VraS mutations or overexpression in isolated S. aureus strains that are resistant to one or more antibiotics. Vancomycin, daptomycin, and telavancin increased *vraSR* gene expression in clinical isolates (6–8). On the other hand, the disruption of *vraS* was found to restore the bacterial sensitivity to vancomycin (9), oxacillin (10, 11), and daptomycin (12). The inactivation of VraS also caused a clinically significant decrease in resistance levels to β-lactam antibiotics, such as methicillin, ceftizoxime, and vancomycin (13). VraS inhibitors were shown to extend or restore the efficacy of some clinically used antibiotics (14). This system is an attractive therapeutic target, as similar histidine kinase signaling circuits are absent in human cells. Thus, VraS inhibitors are expected to have low toxicity (15).

VraS exists as a receptor dimer on the bacterial periplasmic surface with an intracellular histidine kinase domain (16). The N-terminal acts as a signal transfer domain that is theorized to interact with VraT for activation (17). The histidine kinase domain is composed of a dimerization and histidine phosphotransfer domain (DHp) and a catalytic domain (CAT) that binds ATP and catalyzes the phosphorylation and dephosphorylation of the cognate response regulator VraR (18). Upon activation, VraS autophosphorylates its histidine residue H156, which is located in the DHp, and this is followed by transphosphorylation to VraR, which leads to its activation and DNA binding. Phosphorylated VraR positively regulates the cell wall stimulus gene cluster that promotes cell wall thickening and can drive subsequent resistance to antibiotics (19).

There are few reports studying VraS activity *in vitro*, its interaction with VraR, and its inhibition (14, 20). Yet, the factors affecting the kinase activity, the catalytic efficiency, the kinetics of substrate affinity, and the mechanism of inhibition were not analyzed. These aspects are highly relevant to understanding the enzyme functions and designing appropriate *in vitro* assays for inhibitor screening. In this work, we studied the enzymatic activity of a GST-tagged VraS intracellular domain construct encompassing DHp and CAT (GST-VraS) to determine the kinetics of the ATPase reaction and understand the factors that affect activity. We characterized NH125 as a VraS inhibitor and described its effect on the bacterial growth kinetics and the gene expression levels of the S. aureus Newman strain in the absence and presence of cell wall-targeting antibiotics.

## RESULTS

### GST-VraS ATPase rate increases with temperature and in the presence of VraR.

GST-VraS (amino acids 85 to 347) was expressed as previously reported (20). The response regulator binding partner VraR (amino acids 1 to 209) was expressed with a Strep II tag to test its effect on the ATPase reaction rate and inhibition. The purified proteins’ purity values were >90%, and their identities were confirmed by Western blots (WB), using tag-specific antibodies (Fig. S1). A well-established coupled kinase kinetic assay was used to assess the rate of the ATPase activity of GST-VraS at different temperatures and in the absence or presence of the binding partner VraR. The reaction in the absence of VraR represents the autophosphorylation and dephosphorylation of VraS, and, after the addition of VraR, the phosphotransfer and dephosphorylation of VraR were also represented.

**FIG 1:**
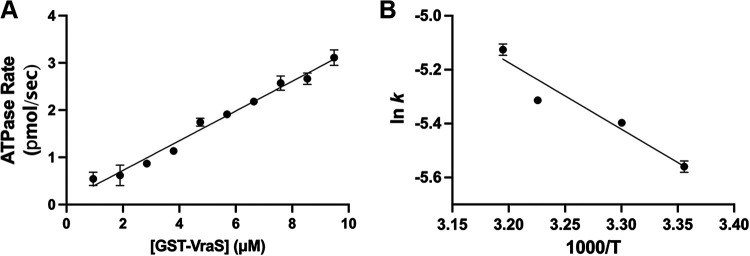
(A) GST-VraS ATPase reaction rate at 22°C, using different enzyme concentrations. (B) Arrhenius plot for the ATPase reaction under different temperatures (25 to 40°C) with 4.5 μM GST-VraS. The data represent the mean ± SD (*n* = 3).

**FIG 2:**
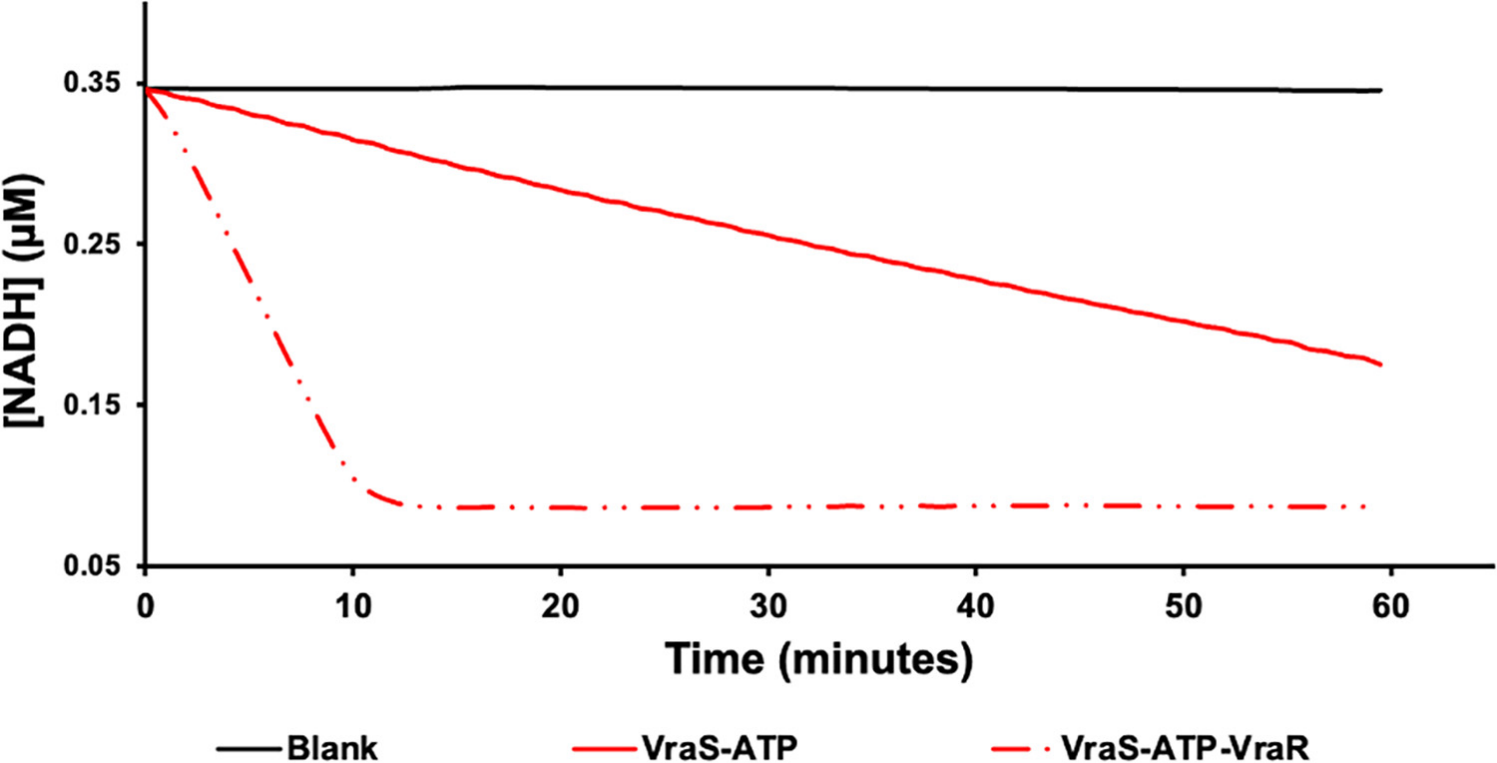
The GST-VraS (4.5 μM) ATPase reaction with and without a 3-molar excess of VraR as a function of the rate of NADH disappearance, compared to a blank reaction. The reaction reaches a plateau with VraR as the NADH in the assay mixture is depleted.

Briefly, the reaction and conversion of ATP to ADP is coupled to a pyruvate kinase/lactate dehydrogenase (PK/LDH) system that converts NADH to NAD^+^. The signal monitored is the decrease in NADH absorbance at 340 nm, which was shown to correspond to the coupled ATPase reaction (21–23). The rate was found to increase with increasing enzyme concentrations (0.95 to 9.49 μM) and temperature (22 to 40°C) (Fig. 1A). Increasing the temperature to 45°C resulted in a non-linear increase of the reaction rate with concentration, indicating the instability of the reaction mixture under these conditions. The GST-VraS activation energy (Ea) for the autophosphorylation reaction was 20.8 ± 1.2 kJ/mol, as determined from the slope (−Ea/R) of the Arrhenius plot (Fig. 1B) derived from the natural logarithm of the Arrhenius equation:

lnk=lnA − Ea/RT,

where *k* is the reaction rate constant, *T* is the absolute temperature, *A* is the preexponential constant, and *R* is the universal gas constant. At 4.5 μM GST-VraS, the reaction rates were found to increase by 3.8-fold in the presence of a 3-molar excess of VraR at room temperature (Fig. 2).

### Magnesium is the preferred divalent metal ion (Me^2+^) for the Me^2+^–ATP substrate complex.

**FIG 3:**
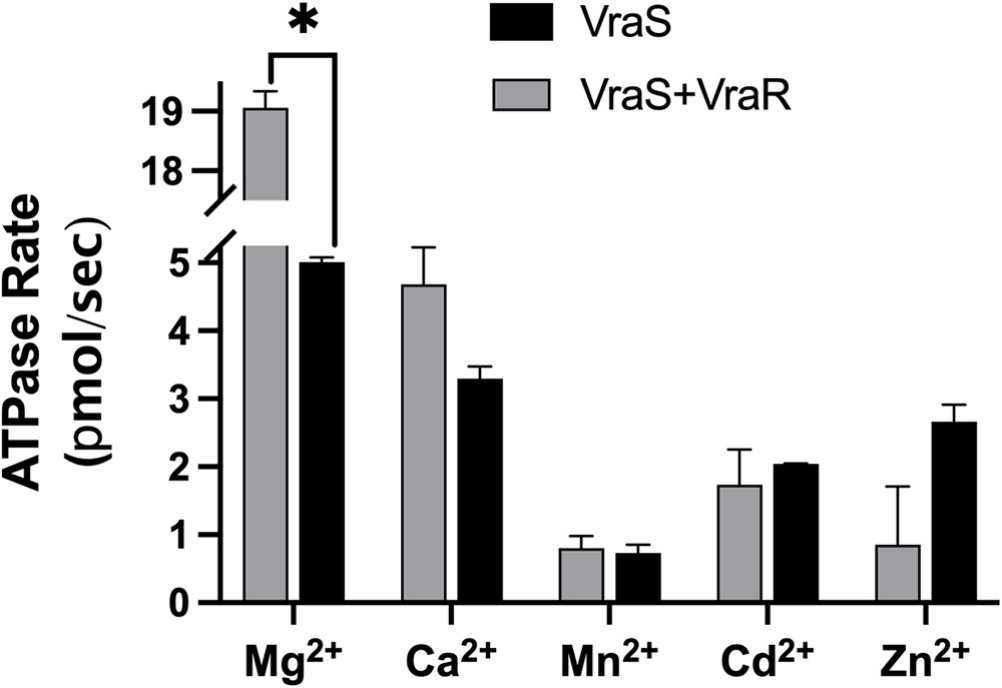
The GST-VraS (4.5 μM) ATPase reaction rates were tested using 10 mM MgCl_2_, CaCl_2_, MnCl_2_, CdCl_2_, or ZnCl_2_ in the presence and absence of an equimolar concentration of VraR. The data represent the mean ± SE (*n* = 3), and statistical significance was calculated via a paired Student’s *t* test (*P* < 0.05).

ATP binding to kinases requires the presence of a divalent cation as the enzyme-substrate complex is formed, and Mg^2+^ is the most commonly utilized divalent cation for histidine kinases (24). We tested 10 mM physiologically and toxicologically relevant metals (Mg^2+^, Ca^2+^, Mn^2+^, Zn^2+^, and Cd^2+^) for their ability to sustain the ATPase reaction in the presence and absence of an equimolar concentration of VraR (25). The autophosphorylation reactions proceeded at different rates with the tested metal ions, with Mg^2+^ supporting the highest reaction rate. The presence of VraR caused a statistically significant 3.8-fold increase in the reaction rate with only Mg^2+^, suggesting its optimal utilization for the ATPase reaction (Fig. 3). The reaction was tested under different MgCl_2_ concentrations (0 to 10 mM), and the maximum catalytic rates were attained at 1 mM MgCl_2_ (Fig. S2). The addition of 10 mM EDTA to the reaction mixture resulted in the abrogation of the signal, indicating the necessity of the presence of divalent cations (results not shown).

### The affinity to ATP is unchanged in the presence of VraR.

**FIG 4:**
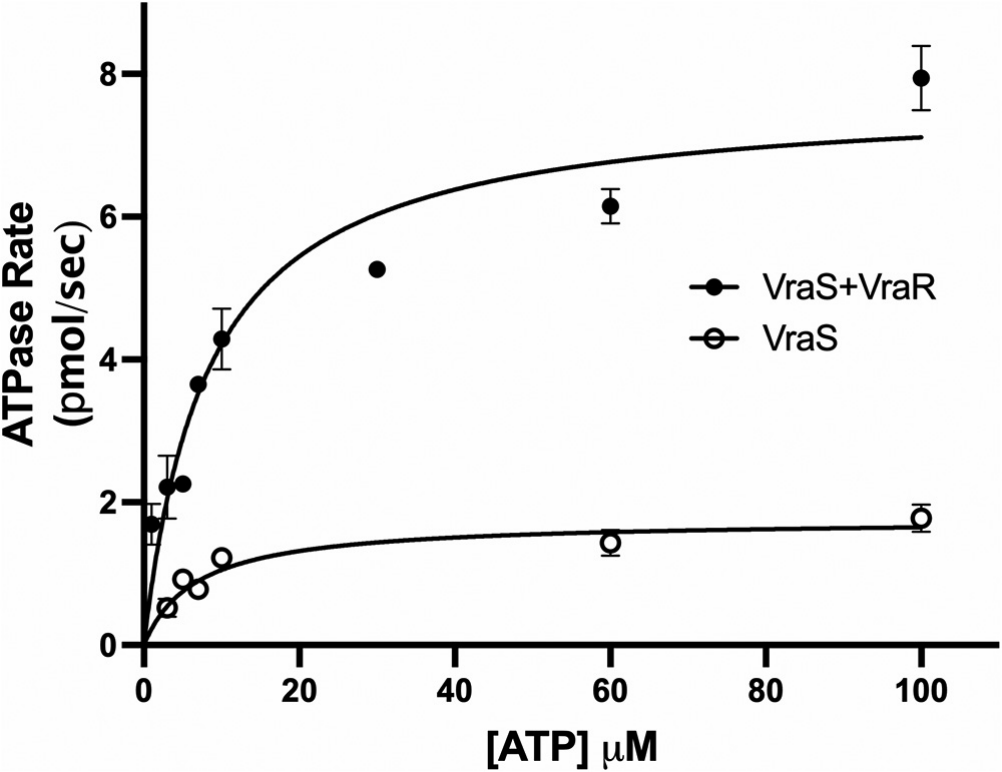
The *K_M_* of ATP was assessed for 1.46 μM GST-VraS with and without a 3-fold molar excess of VraR. The solid lines represent the nonlinear regression curve that was fit to the Michaelis-Menten equation. The data represent the mean ± SE (*n* = 2 biological replicates of three technical measurements).

To determine the kinetic parameters of ATP binding to GST-VraS, the reaction rate was monitored at variable ATP concentrations (1 to 100 μM), using 1.46 μM GST-VraS in the presence and absence of a 3-fold molar excess of VraR. GraphPad Prism was used to determine the kinetic parameters by fitting the data to the Michaelis-Menten kinetics equation:

$$v_{0}=\frac{V_{\max\;}[S]}{K_{M}+[S]},$$

where $v_{0}$ is the initial rate of the reaction, *V_max_* is the maximum ATPase reaction rate from which the apparent catalytic rate (*k_cat_*) can be calculated, [S] is the substrate (ATP) concentration, and *K_M_* is the apparent Michaelis constant (Fig. 4). The addition of VraR did not change the affinity of GST-VraS to ATP, as represented by the insignificant change in the apparent *K_M_* of the reaction, while *k_cat_* had significantly increased by 4.1-fold (Table 1). The increase in the catalytic rate can be attributed to the continuously replenished unphosphorylated pool of VraS after the phosphotransfer reaction proceeded in the presence of VraR.

**TABLE 1:** The kinetic parameters of the GST-VraS (1.46 μM) ATPase reaction in the absence and presence of VraR^*a*^

VraR	$K_{M}$ of ATP (μM)	$V_{\max}$ (pmol sec^−1^)	$k_{\mathrm{cat}}$ (10^−3^ s^−1^)	$k_{\mathrm{cat}}/K_{M}$ (mM^−1^ s^−1^)
Absence	6.7 ± 1.6	1.8 ± 0.1	16.9 ± 5.6	2.6 ± 0.6
Presence	8.3 ± 1.9	7.7 ± 0.5	69.81 ± 0.01	8.7 ± 1.9

a The data represent the mean ± SE (*n* = 2 biological replicates of three technical measurements).

### NH125 inhibits GST-VraS in a noncompetitive manner with attenuated inhibition in the presence of VraR.

**FIG 5:**
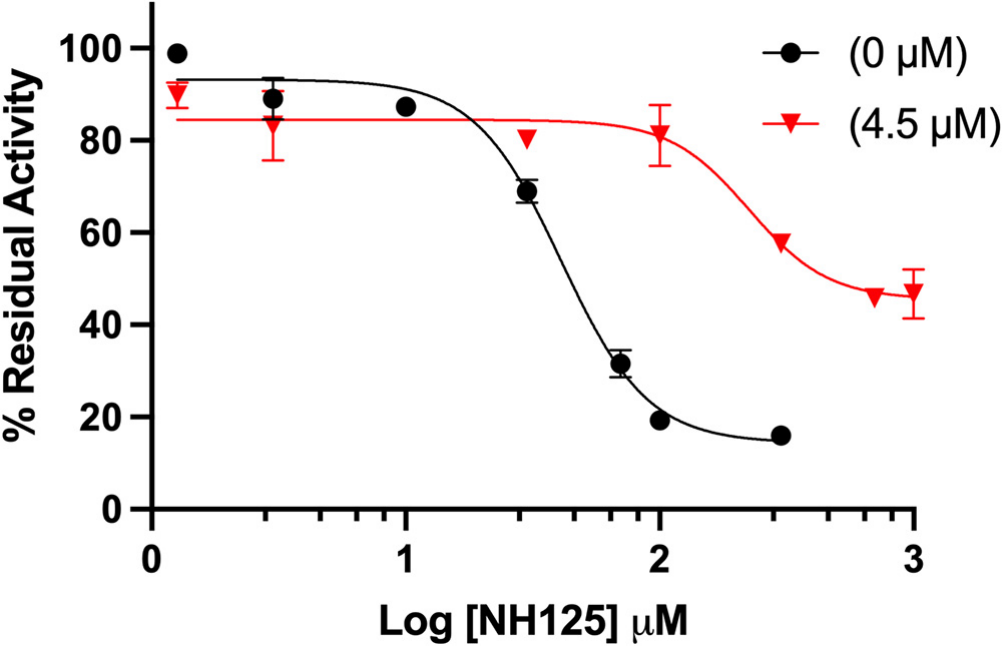
The inhibition of GST-VraS (4.5 μM) ATPase activity by increasing the concentrations of NH125 in the absence or presence of equimolar concentrations of VraR. The data represent the mean ± SD (*n* = 3).

**FIG 6:**
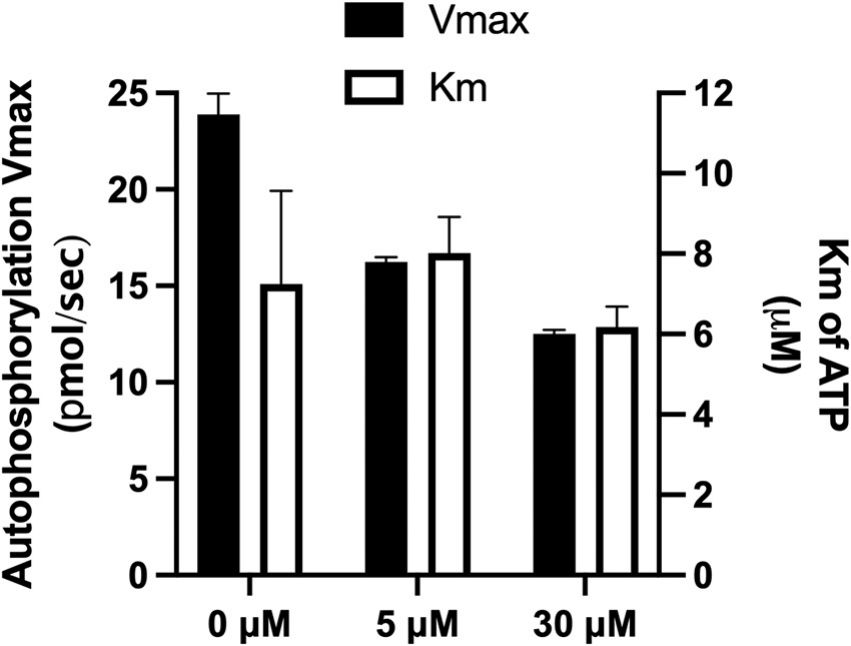
The effect of different concentrations of NH125 on the kinetic parameters of GST-VraS (10 μM) autophosphorylation. The data represent the mean ± SE (*n* = 2 biological replicates of three technical measurements).

NH125 is a known kinase inhibitor that was reported to thwart the growth of drug-resistant bacteria by inhibiting several histidine kinases (26–28). We tested the activity of GST-VraS (4.5 μM) in the presence of increasing concentrations of NH125, and the results indicate the compound’s ability to inhibit the enzyme with an IC_50_ of 41 ± 5 μM. Adding an equimolar concentration of VraR to the reaction mixture led to a significant 7.3-fold increase of the IC_50_ value to 298 ± 36 μM (Fig. 5). We conducted Michaelis-Menten kinetics studies to understand the mechanism of inhibition of GST-VraS (10 μM) in the presence of fixed NH125 concentrations (0 to 30 μM). There was no change in the *K_M_* of ATP, while the *V_max_* of the reaction showed a gradual decline, indicating that the inhibition is noncompetitive in nature (Fig. 6).

### NH125 affects the S. aureus growth kinetics and decreases the gene expression of *vraSR*, *blaZ*, and *pbpB* in the presence of antibiotics.

**FIG 7:**
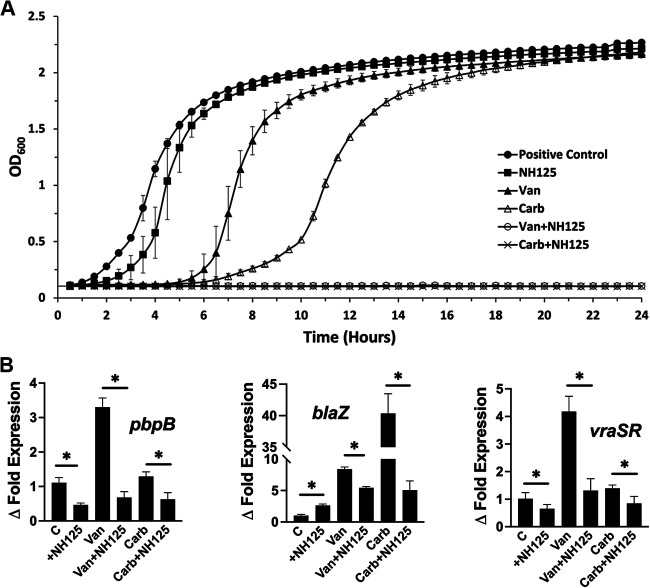
The effect of the addition of 50 μM NH125 to the S. aureus Newman strain in the presence and absence of carbenicillin (Carb) and vancomycin (Van) on (A) bacterial growth kinetics, relative to the positive control (no antibiotics, no NH125), and (B) the fold change in the expression levels of *vraSR*, *pbpB*, and *blaZ*, relative to the positive-control culture (C). The data represent the mean ± SD (*n* = 3), and statistical significance was calculated via a paired Student’s *t* test (*P* < 0.05).

We tested the effect of NH125 inhibition on the growth kinetics of the S. aureus Newman strain in the presence and absence of cell wall-targeting antibiotics. The MICs of carbenicillin and vancomycin were determined to be 2.34 and 0.38 μg/mL, respectively, using the broth microdilution method, according to European Committee on Antimicrobial Susceptibility Testing (EUCAST) guidelines. The antibiotics were added to cultures at 0.5 MIC to allow for sufficient growth under a stress signal, and the growth kinetics were monitored over 24 h, as detailed in Materials and Methods. In the absence of antibiotics, the addition of 50 μM NH125 did not alter the growth rate significantly, whereas the addition of antibiotics led to an increase in the lag phase duration and lower slopes of growth in the exponential phase. The combination of NH125 and antibiotics at these sublethal doses led to the complete abrogation of bacterial growth, indicating its effectiveness as a resistance-modifying agent (Fig. 7A). We assessed the effect of NH125 inhibition on the expression levels of the *vraSR* gene, the *pbpB* gene controlled by VraS that translates to a PBP2 transpeptidase that is required for cell wall formation, and the *blaZ* gene that translates to the β-lactamase enzyme, both of which are involved in bacterial resistance (29). Cultures were grown to an OD_600_ of approximately 0.6 and treated for 1 h with 50 μM NH125 with or without the antibiotics at the 2× MIC level, as detailed in Materials and Methods. The gene expression was normalized to determine the relative expression, and the fold change in expression was compared to a control culture. The addition of NH125 caused a decrease in both *vraSR* and *pbpB* expression, consistent with its inhibition of the basal expression of VraS. However, there was an increase in *blaZ* expression. While the addition of vancomycin to the culture caused a significant increase in the expression of all genes as previously reported (30, 31), carbenicillin increased only the *blaZ* expression. The addition of NH125 with both antibiotics led to a significant reduction in gene expression for all three genes, compared to the cultures with antibiotics only (Fig. 7B).

## DISCUSSION

Two-component systems have received attention for their important roles in modulating bacterial defense systems implicated in developing antibiotic resistance. Unfortunately, the discovery rate of new antibiotics targeting resistant strains is slower than the rate of resistance development. This prompted a search for resistance-modifying agents that can extend or restore the efficacy of existing antibiotics. The biochemical analysis and characterization of the VraS sensor histidine kinase allow for a better understanding of enzymatic activity and modulation. GST-VraS can reliably be used to characterize the enzyme because, aside from signal transfer, the processes of VraS dimerization, catalytic activity, and VraR binding are all represented by this construct. The same construct was used in multiple studies to study VraS (14, 20), and a similar approach has been adopted with several histidine kinases to overcome the insolubility of the full-length protein (32–37).

The activity of GST-VraS increased linearly with increasing enzyme concentrations and temperature with an Ea of 20.8 ± 1.2 kJ/mol, which is close to the reported values of bacterial adenylate kinase (16.9 ± 0.5 kJ/mol), acetate kinase (13 to 16 kJ/mol), and phosphomevalonate kinase (23.4 kJ/mol) (38–40). Metal ions (Me^2+^) are needed for ATP binding to kinases to neutralize the charge and adequately orient the polarized γ-phosphoryl group, which facilitates the phosphorylation reaction (41). Many divalent metal ions can be utilized for ATP binding, but Mg^2+^ is considered to be the physiological activator *in vivo* due to its high concentration in the cell (42). GST-VraS autophosphorylation activity favored Mg^2+^ as the divalent cation for the Me^2+^-ATP substrate complex, similar to MtrB and WalK, which are sensor kinases in Mycobacterium tuberculosis and Streptococcus pneumoniae (35, 43). Maximum activity was reached at approximately 1 mM Mg^2+^, which is much lower than the estimated intracellular levels in mammalian and bacterial cells (44). Adding single or combined monovalent cations did not increase the reaction rate significantly (Fig. S3), unlike the reported activation of other histidine kinases, such as EnvZ and CheA (45, 46).

In the presence of VraR, the rate of the GST-VraS ATPase reaction shows a 3.8-fold increase that can be attributed to an increased pool of recycled dephosphorylated kinase after the phosphoryl transfer to VraR. The results may also indicate that the binding of VraR to one subunit of the GST-VraS dimer may lead to conformational changes in the other subunit that enhance its autophosphorylation. Further investigation is needed to clarify the exact cause of that increase. The *K_M_* of ATP was 6.7 ± 1.6 μM, which is below the cellular millimolar ATP concentration, indicating the intracellular saturation of VraS with the nucleotide. The value is within the range of the ATP affinity to other sensor kinases, such as NarX (2.4 ± 0.7 μM), PhoQ (17.7 ± 1.4 μM), NarQ (22.8 ± 9.3 μM), and WalK (42.0 ± 2.2 μM) (35, 36, 47). There was no significant difference between the *K_M_* of ATP in the presence or absence of VraR, which was expected, based on TCS molecular interaction models (48). It is possible that the autophosphorylation of VraS dimeric units may be cooperative in nature. Unfortunately, due to the low VraS activity at lower ATP concentrations, it was not clear whether the early parts of the curves showed a noncooperative (hyperbolic) or cooperative (sigmoidal) fit. Further investigation is needed to distinguish between the two possibilities.

We tested the effect of NH125, which is a known histidine kinase inhibitor, on the activity and catalytic rate of GST-VraS. The compound inhibited the kinase activity with an IC_50_ of 41 ± 5 μM that was attenuated in the presence of VraR. The tracking of the effect of increasing concentrations of NH125 on the catalytic rate and substrate affinity indicated that NH125 inhibited VraS via a noncompetitive mechanism in which the inhibitor binds to an allosteric site on the free enzyme that is distinct from the ATP binding site, similar to other reported histidine kinase inhibitors (49). The decrease in inhibition in the presence of VraR may reflect the increase in the catalytic efficiency of the enzyme in the presence of its cognate response regulator or may indicate an overlap between VraR and NH125 binding sites on VraS that warrants further investigation.

The S. aureus cultures showed an expected decrease in growth kinetics in the presence of sublethal doses of the cell wall-targeting antibiotics carbenicillin and vancomycin. The growth rate was not affected by NH125, which is supported by the nonessentiality of TCS in S. aureus (except for WalRK) growing under replicating conditions (50). The combination of the antibiotic sublethal stress signal and the inhibition of VraS led to the complete abrogation of the bacterial growth, indicating a synergistic effect between the antibiotics and NH125. While qRT-PCR results indicated that the gene expression levels of *pbpB* and *vraSR* were significantly decreased after adding NH125 to Newman cultures, *bla*Z expression showed an almost 2-fold increase. This may indicate that NH125 upregulates *blaZ* through a different mechanism, which warrants further investigation. The addition of vancomycin caused an expected increase in the expression of all three genes, which is consistent with published data, but the addition of carbenicillin caused only an increase in *blaZ* expression. This result cannot be attributed to the difference in antibiotic classes, as oxacillin (a β-lactam like carbenicillin) was also reported to increase the expression of *vraSR* and *pbpB*, at least in MRSA strains (10, 13, 31). The addition of antibiotics in the presence of NH125 caused a significant decrease in the expression of all three genes, compared to cultures with antibiotics only, indicating that the inhibition of VraS affects downstream genes that are related to antibiotic resistance and are controlled by this critical histidine kinase.

Knowledge of the kinetic parameters of VraS is vital to design appropriate assays with ATP concentrations that will allow for the detection of new competitive inhibitors. The results show a decrease in the inhibitor’s potency in the presence of the natural substrate, pointing to the importance of including the cognate response regulators in screening assays to discover histidine kinase inhibitors. The absence of the natural substrate may lead to the discovery of compounds that show less efficiency in microbiological testing and have lower translational potential as resistance-modifying agents. The growth kinetic curves and the levels of downstream gene expression indicate the efficacy of NH125 as a VraS inhibitor as well as its potential as a resistance-modifying agent.

## MATERIALS AND METHODS

### Materials, reagents, and plasmids.

All of the chemicals were obtained from Fisher Scientific unless otherwise stated. The LB/carbenicillin plates, Tris-Glycine-SDS, and TBST buffers were from Teknova. The gels and Trans-Blot Turbo Ready-To-Assemble (RTA) Mini 0.2 μm PVDF Transfer Kits were from Bio-Rad. GST-VraS and Strep-VraR were probed in WB with GST Tag Antibody, HRP Conjugate (Invitrogen number MA4-004-HRP), and Strep-Tag II Antibody HRP Conjugate (EMD Millipore number 71591-M), respectively. For the kinase assay, the PK/LDH mixture was from Sigma, and the 96-well plates were from Corning (number 3695). The constructs of GST-VraS (UniProt accession number Q99SZ7) in a pGEX-4T-1 plasmid and Strep-VraR (UniProt accession number Q7A2Q1) in a p51b plasmid were generous gifts from Aurijit Sarkar.

### Protein expression and purification.

The proteins were expressed in BL21(DE3) cells, per published protocols (20). For protein purification, the cell pellets were resuspended in lysis buffer (50 mM Tris HCl [pH 8], 1 mM EDTA for GST-VraS, as well as 100 mM Tris HCl [pH 8], 150 mM NaCl, 10% (vol/vol) glycerol, 1 mM EDTA, and 0.01% (vol/vol) Tween for VraR) that was freshly supplemented with 1 mM PMSF and protease inhibitor cocktail tablets as well as lysed in a microfluidizer (Microfluidics), using two passes under 15,000 lb/in^2^. The cell lysate was clarified via centrifugation at 12,298 × *g* for 1 h at 4°C. The supernatant was loaded onto a Bio-Rad FPLC system that was equipped with either 1 mL GSTrap HP or 1 mL StrepTrap HP columns (GE Healthcare Life Sciences) for GST-VraS and VraR, respectively. The protein was eluted via gradient elution over 10 column volumes, using elution buffer (lysis buffer supplemented with 10 mM reduced glutathione or 2.5 mM d-desthiobiotin for GST-VraS and VraR, respectively). The fractions containing the protein were pooled and concentrated, and the buffer was exchanged into lysis buffer using Amicon Ultra Centrifugal Filters before allocation and freezing at −80°C. The protein concentration was determined via a 660 nm protein assay (Pierce), using bovine serum albumin as the reference standard. The purities and identities of the target proteins were estimated via SDS-PAGE and WB, using tag-specific antibodies. Chemiluminescence detection was done using Thermo Scientific Pierce ECL Substrate, per the manufacturer’s protocol.

### Coupled kinase assay and kinetic parameters.

The enzymatic reactions were conducted at room temperature with a mixture containing 20 mM Tris [pH 7.5], 2 mM ATP, 10 mM MgCl_2_, 55 U/μL PK/LDH mix, 1 mM phosphoenol pyruvate, and 0.35 mM NADH, unless otherwise indicated in Results. The assay mixture and the enzyme were incubated for 10 min under the testing temperature. The reaction rate was monitored over time, and the early linear slope of the reaction (theoretical 10% substrate consumption) was used to calculate the kinetic parameters. The resulting absorbance was mathematically transformed to concentration using the NADH extinction coefficient (6,220 L mol^−1^ cm^−1^), and the rate of the reaction was calculated from the rate of product formation, per published protocols (51). All curve fittings for kinetic parameters were conducted with the Michaelis-Menten equation, as implemented in GraphPad Prism, version 9.4.1 (GraphPad Software, San Diego, USA). Standard controls were applied to verify that the reaction rate is attributed to GST-VraS activity. This includes doubling the enzyme concentrations (resulting in the doubling of the rate) and eliminating GST-VraS or using a mock purification product after transformation with an empty plasmid (resulting in signal abrogation) and doubling the LDL/PK supporting enzymes or the phosphoenol pyruvate (no effect on rates).

### Bacterial growth, RNA extraction, and qRT-PCR.

Cultures of the S. aureus Newman D2C strain (ATCC number 25904) were diluted from overnight cultures to an OD_600_ of approximately 0.1 and grown for 24 h with or without NH125, carbenicillin, or vancomycin, as indicated in Results. Growth was monitored over 24 h by measuring the optical density at 600 nm (OD_600_) using a microplate reader (Synergy HT) with continuous shaking at 37°C. For the qRT-PCR, the cultures were grown until an OD_600_ of approximately 0.6 at 37°C. Then, antibiotics at the 2× MIC level were added with or without NH125 for 1 h before harvesting. The total RNA was extracted by resuspending pellets in lysozyme (0.4 mg/mL) and by using a GeneJET RNA Purification Kit (Thermo Scientific). cDNA was synthesized using a SuperScript VILO cDNA Kit (Invitrogen), according to the manufacturer’s protocols. The gene expression levels were determined via qRT-PCR, using a QuantStudio 5 real-time PCR system with Power Track Syber Green Master Mix (Applied Biosystems). The gene of the ribosomal protein L4, namely, *rplD*, was used as a reference gene for normalization to track the expression levels of the *vraSR*, *pbpB*, and *blaZ* genes (the sequences of the primers that were used are listed in Table S1). After normalization, we used the 2^−ΔΔCT^ method to calculate the relative fold changes in the gene expression levels, relative to the untreated control.

### Data availability.

This published article and its supplementary material include all of the data that were generated during this study.
