# Supplementary material for: NH125 Sensitizes Staphylococcus aureus to Cell Wall-Targeting Antibiotics through the Inhibition of the VraS Sensor Histidine Kinase
Source: Microbiol Spectr. 2023 May 25;11(3):e04861-22. doi: 10.1128/spectrum.04861-22 (PMC10269531; doi:10.1128/spectrum.04861-22)
Supplement: Supplemental file 1 — Supplemental material. Download spectrum.04861-22-s0001.pdf, PDF file, 0.3 MB [file spectrum.04861-22-s0001.pdf]

NH125 Sensitizes *Staphylococcus aureus* to Cell Wall Targeting Antibiotics through the Inhibition of VraS Sensor Histidine Kinase

Shrijan Bhattarai[1], Lane Marsh[1], Kelsey Knight[1], Liaqat Ali[1], Antonio Gomez[1], Allison Sunderhaus[1], and May H. Abdel Aziz[1#]

[1]Fisch College of Pharmacy, The University of Texas at Tyler, Tyler, TX, USA.

Running Title: NH125 Inhibition of VraS

[#] Address correspondence to: mabdelaziz@uttyler.edu

Supplementary Figure 1

a) GST-VraS and **b)** VraR purification: Left panels represent the SDS-PAGE of the purifications (M: molecular marker, L cell lysate, F column flow through, W wash and E, fractions eluted). Right panels are Western blots for the same gels to confirm protein identity using tag specific antibodies as indicated in the Materials section.

a)

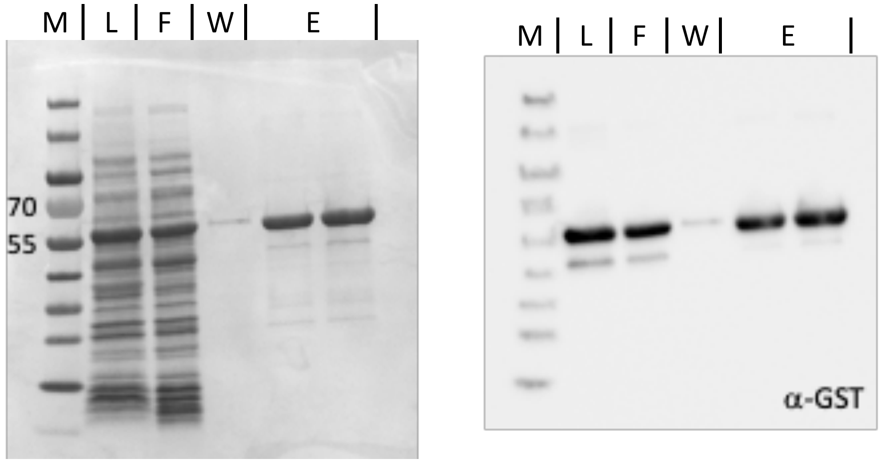

b)

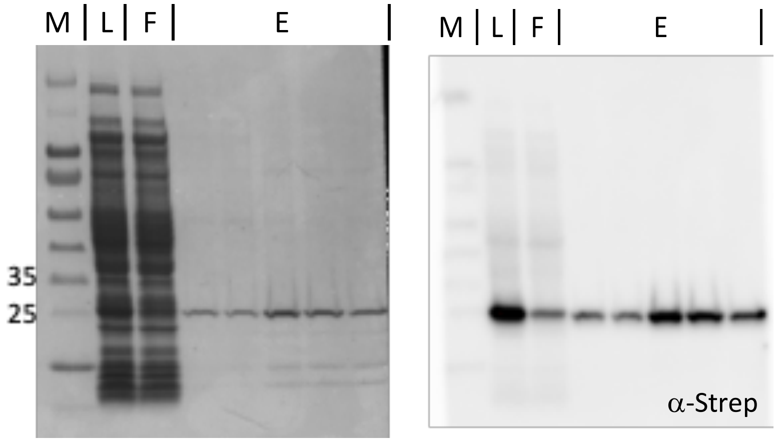

Supplementary Figure 2

The autophosphorylation reaction rates of GST-VraS (4.5 μM) at room temperature using increasing concentrations of $MgCl_2$.

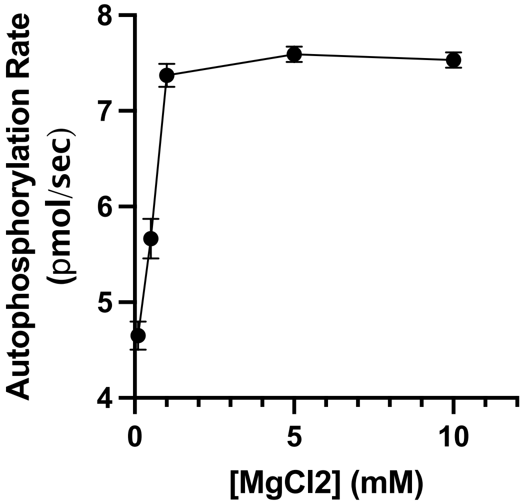

Supplementary Figure 3

The effect of adding 25 mM total concentration of monovalent cations to the reaction mixture on GST-VraS autophosphorylation rates. The data represent the mean ± S.E (n = 2). The statistical significance was calculated using a paired Student t-test ($P < 0.05$).

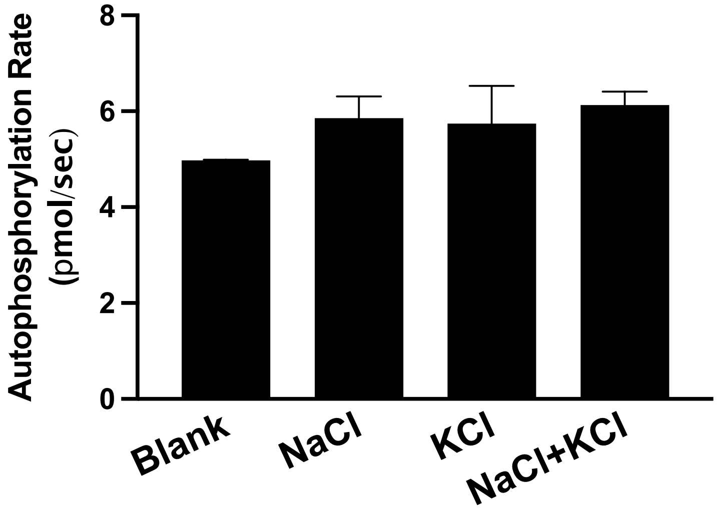

Supplementary Table 1

qRT-PCR primers used to amplify *S. aureus* genes. Primer sequences are written 5′ to 3′. Please see Materials and Methods for details.

Gene	Forward Primer	Reverse Primer	Reference
pbpB	CAAGCAACAGATCCTCACCCT	AATCGCATGGTTTGTTGCCC	Gillard et al.
rplD	TTCGGACCAACTCCAAGA	CGAGCACCTCCTCAAC	Sihto et al.
vraR	GCGCGCTTTTTCATACGGTT	ATCGCCGATGCAGTTCGTAA	Cutrona et al.
blaZ	GCTTTAAAAGAACTTATTGAGGCTTCA	CCACCGATYTCKTTTATAATTT	Pereira et al.

Reference:

Cutrona, N., Gillard, K., Ulrich, R., Seemann, M., Miller, H. B., & Blackledge, M. S. (2019). From antihistamine to anti-infective: loratadine inhibition of regulatory PASTA kinases in Staphylococci reduces biofilm formation and potentiates β-lactam antibiotics and vancomycin in resistant strains of *Staphylococcus aureus*. ACS Infect. Dis., *5*(8), 1397-1410.

Gillard, K., Miller, H. B., and Blackledge, M. S. (2018) Tricyclic amine antidepressants suppress beta-lactam resistance in methicillin-resistant *Staphylococcus aureus* (MRSA) by repressing mRNA levels of key resistance genes, Chem Biol Drug Des 92, 1822-1829.

Pereira, L. A., Harnett, G. B., Hodge, M. M., Cattell, J. A., and Speers, D. J. (2014) Real-time PCR assay for detection of *blaZ* genes in *Staphylococcus aureus* clinical isolates, J Clin Microbiol 52, 1259-1261.

Sihto, H. M., Tasara, T., Stephan, R., & Johler, S. (2014). Validation of reference genes for normalization of qPCR mRNA expression levels in *Staphylococcus aureus* exposed to osmotic and lactic acid stress conditions encountered during food production and preservation. FEMS Microbiol. Lett., *356*(1), 134-140.
